# Autologous osteochondral transplantation provides succesful recovery in patients with simultaneous medial and lateral talus osteochondral lesions

**DOI:** 10.5152/j.aott.2021.21204

**Published:** 2021-11-01

**Authors:** Çağrı Örs, Yaman Sarpel

**Affiliations:** Clinic of Knee and Sport Surgery, Private Ortopedia Hospital, Adana, Turkey

**Keywords:** Osteochondral lesion of the talus, Autologous osteochondral transplantation, Simultaneous medial and lateral OLT, Medial malleolar osteotomy, Distal fibular osteotomy

## Abstract

**Objective:**

The aim of this study was to evaluate the success of simultaneous medial and lateral autologous osteochondral transplantation (AOT) in patients with both medial and lateral osteochondral lesions of the talus (OLT) who could not be treated with bone marrow stimulation techniques or whose previous treatments failed.

**Methods:**

Five patients who underwent medial and lateral talus AOT in the same session due to simultaneous OLT on medial and lateral talar dome were included in this retrospective study. All the patients were male, and the mean age was 34.8 (range = 26 – 42) years. Double osteochondral grafts were placed separately to the medial and lateral talar dome at the same session. Location and size of the OLT, additional ligament injuries, and postoperative evaluation of the graft were evaluated through magnetic resonance images (MRIs). The criteria of Berndt and Harty, Single Assessment Numeric Evaluation (SANE), American Orthopedic Foot and Ankle Society Score (AOFAS), visual analogue scale (VAS) score, and Tegner Activity Score were assessed preoperatively and at the last follow-up.

**Results:**

All patients had suffered an injury with inversion of the foot while daily or sports activity. Average duration of symptoms from onset to osteochondral grafting was 25.2 months. The mean follow-up time was 61 months. The radiological evaluation showed OLT with Stage 4 or 5, anterior talofibular ligament injury and deltoid ligament strain observed in all of the patients. With the numbers available, pre and post-operative comparisons showed a significant increase in patients’ satisfaction rate and significant decrease in VAS scores as well as an increase in AOFAS scores with the numbers available. No complications were encountered.

**Conclusion:**

Simultaneous AOT for medial and lateral OLT appears to be a reliable surgical procedure with satisfactory clinical results.

**Level of Evidence:**

Level IV, Therapeutic Study

## Introduction

Osteochondral Lesions of the Talus (OLT) are common injuries, which are known as symptomatic lesions causing pain, recurrent synovitis, altered joint mechanics in ankle joint and also acting as a precursor of ankle osteoarthritis.^1-^^3^ Symptomatic OLT often requires surgical treatments with a variety of methods to diminish symptoms like pain and swelling, to improve function and return to activity before the injury.^1-^^3^ Surgical treatments can be divided into two groups. The first one is the reparative Bone Marrow Stimulation (BMS) procedures such as arthroscopic microfracture or drilling and the second one is the placement procedures such as Autologous Osteochondral Transplantation (AOT) or chondrocyte transplantation.

AOT procedure is often indicated for symptomatic large OLT and cystic lesions (stage 5) as well as for lesions that have failed previous reparative procedures, such as BMS techniques.^1-^^5^ The perceived advantages of AOT are to replace a talar lesion with hyaline cartilage with a subchondral bone plug that is native to the host.^6^ A recent systematic review of clinical outcomes demonstrated good/excellent outcomes in 87% of patients treated with AOT.^7^ However, there is limited evidence in the literature regarding the treatment of patients with simultaneous medial and lateral OLT with AOT.

OLT is seen more frequently on the medial talar dome.^1,3,6^ In contrast, lateral OLT are less common and mostly located anterolaterally, which can be accessed without osteotomy.^6^ However, a particular kind of lesion remains to be studied in terms of treatment, which is the simultaneous medial and lateral OLT. A very important reason for that is the low frequency of simultaneous medial and lateral OLT. In a systemic review evaluating 1361 patients with OLT, only 1% was reported as medial and lateral.^3^

In our opinion, simultaneous medial and lateral AOT should be performed in patients with both medial and lateral OLT where BMS techniques have failed or there are no appropriate indications. We aimed to present and evaluate the treatment results of simultaneous medial and lateral AOT of five patients with both medial and lateral OLT whose previous arthroscopic treatments were not successful.

## Materials and Methods

### Study population

**Figure 1. a-d.**
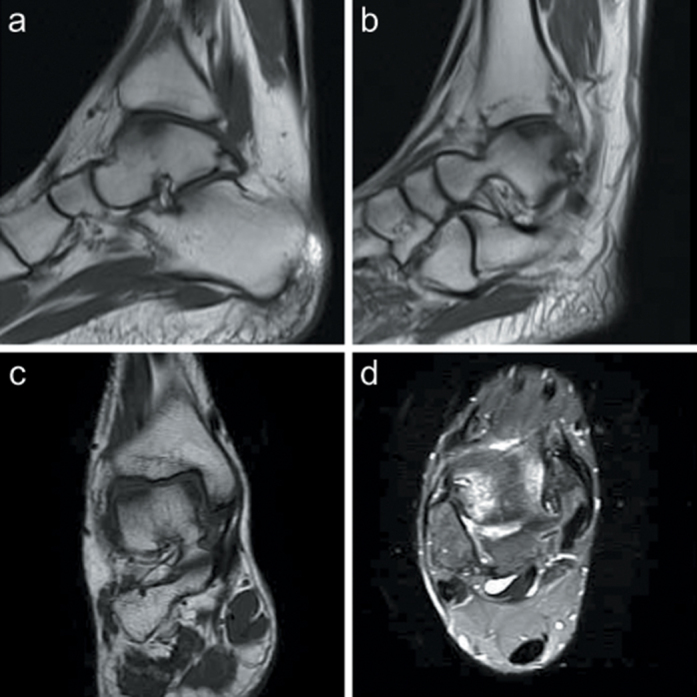
Preoperative magnetic resonance images. (a) Preoperative T1 weighted sagittal magnetic resonance images of OLT on medial talar dome, (b) T1 weighted sagittal magnetic resonance images of OLT on lateral talar dome, (c) T1 weighted coronal magnetic resonance images of OLT on medial and lateral side simultaneously, and (b) T2-weighted axial magnetic resonance images of OLT on medial and lateral talar domes.

**Figure 2. a-d.**
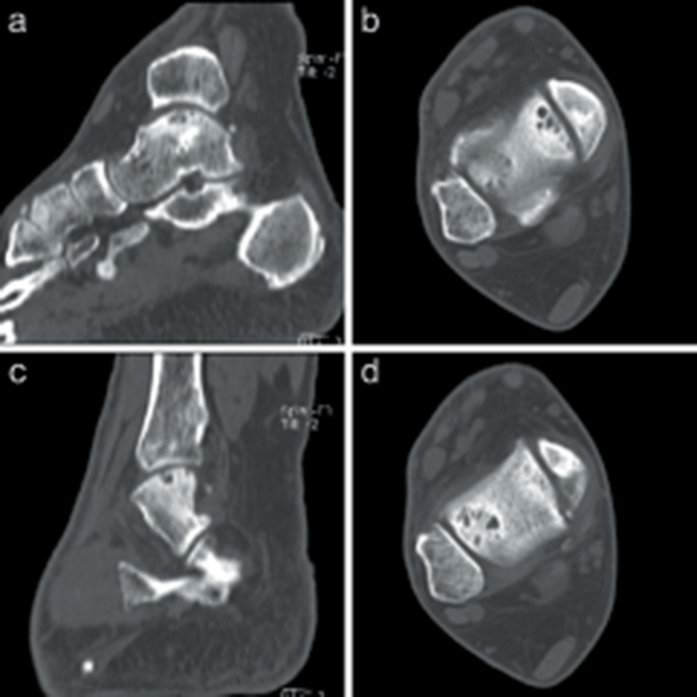
Preoperative computed tomography images. Preoperative (a) sagittal and (b) axial computed tomography images of OLT on medial talar dome. (c) Sagittal and (d) axial computed tomography images of OLT on lateral talar dome.

In this retrospective study, we included five patients who underwent medial and lateral talus AOT in the same session due to simultaneous OLT on medial and lateral talar domes, for which previous arthroscopic debridement and microfracture treatment were not successful and OLTs were not suitable for BMS techniques. The clinical research ethics committee of Çukurova University approved this study (28/107). The mean ± standard deviation (STD) of ages for patients was 34.8 ± 7.2 years (range, 26–42) at the time of surgery and all patients were male. The mean follow-up time was 61 months (range, 32-85). Preoperative ankle MRI was performed (Figure 1) and computed tomography (CT) was performed to evaluate subchondral bone and additional bone pathologies in patients with stage 5 cystic lesions (Figure 2). The following variables were collected: mechanism of injury, duration of injury, additional ligament injury, location and size of lesion on MRI and intraoperatively (Table 1).

**Figure 3.**
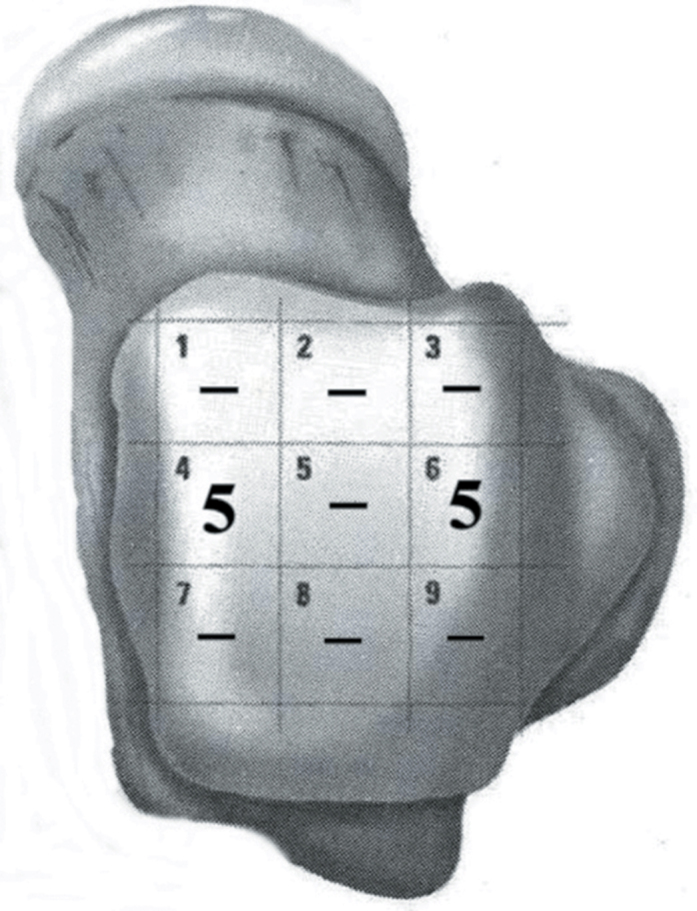
Osteochondral lesions distribution according to the location of the 9 sites articular surface described by Raikin et al.^9^

Osteochondral lesions of the patients in the medial and lateral talar domes were stage 4 or 5 according to Modified Bristol Classification based on MRI.^8^ The following parameters were defined for each patient: coronal length (horizontal extension measured from the coronal image), sagittal length (horizontal extension measured from the sagittal image) and lesion area (as calculated by the ellipse formula, from coronal and sagittal length: A = abπ = coronal length × sagittal length × 0.79). The talar dome lesions were performed according to the location of the nine sites articular surface described by Raikin et al. both on medial and lateral sites (Figure 3).^9^

### Surgical technique

An anteromedial incision was made, starting approximately 4 cm proximal to the ankle joint level, extending distally and slightly anteriorly, and ending approximately 2 cm distal to the tip of the medial malleolus. The medial malleolus was drilled and prepared for screw fixation. A chevron-type medial malleolar osteotomy was performed to reach the medial side of the talar dome. A slightly curved lateral incision was used, starting from 6 cm proximal to the ankle joint level, extending along the anterior of the fibula and in line with the fourth ray distally. The superficial peroneal nerve was protected carefully. Anterolateral arthrotomy was performed. In two of the cases where adequate perpendicular exposure to the talar lesion could not be achieved, distal fibular osteotomy was required. The anterior inferior tibiofibular ligament was incised. Oblique distal fibular osteotomy was performed 3 cm above the joint surface with Anterior Talofibular Ligament (ATFL) release (P2 and P5). The osteotomy was performed obliquely for use of the donor harvesting device vertically easily.

**Figure 4. a-d.**
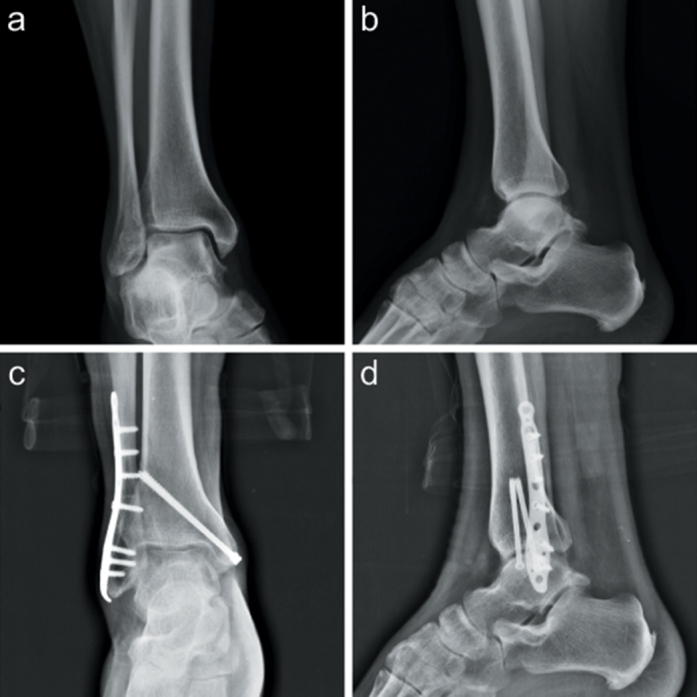
Autologous osteochondral transplantation on medial and lateral talar dome images during surgery. (a) and (b) The view of the autologous osteochondral transplantation on the medial side and medial malleolus is marked with yellow arrow. (c) and (d) The view of the autologous osteochondral transplantation on the lateral side.

The lesions were measured and then removed with the appropriate size recipient cutting tube (6, 8, or 10 mm) to a depth of 12-15 mm for AOT (Single Use OAT System, Arthrex, and Naples, FL, Italy). The osteochondral grafts were harvested from the anterior aspect of the ipsilateral lateral femoral condyle articular facet. A donor harvester device was used (Single Use OAT System, Arthrex, Naples, FL, Italy) and the graft was inserted and gently tamped into the recipient site until it was at the same level as the surrounding cartilage edges. A donor harvester device was used (Single Use OAT System, Arthrex, Naples, FL, Italy) and the graft was inserted and gently tamped into the recipient site until it was at the same level as the surrounding cartilage edges (Figure 4). In the end, the osteotomized medial malleolus was repositioned and fixed with one acutrack headless screw or two cannulated titanium screws. If a lateral malleolar osteotomy was performed, osteotomized lateral malleolus was repositioned and fixed with anatomic distal fibular plate with syndesmosis screw fixation just above the joint level to be removed after 6 weeks.

### Follow-up

The post-operative rehabilitation protocol included no weight-bearing mobilization in a cast for 3 weeks, partial weight-bearing mobilization in a walking boot, physiotherapies for the next 4 weeks, and then full weight-bearing mobilization. Informed consent was obtained from each patient in the study.

**Figure 5. a-d.**
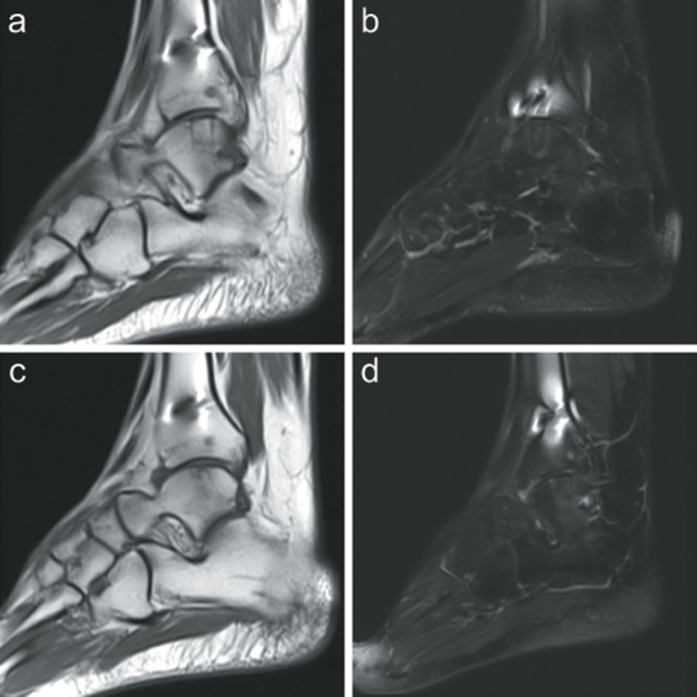
Pre and postoperative radiographs. Preoperative (a) anteroposterior and (b) lateral radiographs. Postoperative 1^st^ year (c) anteroposterior and (d) lateral radiographs.

**Figure 6. a-d.**
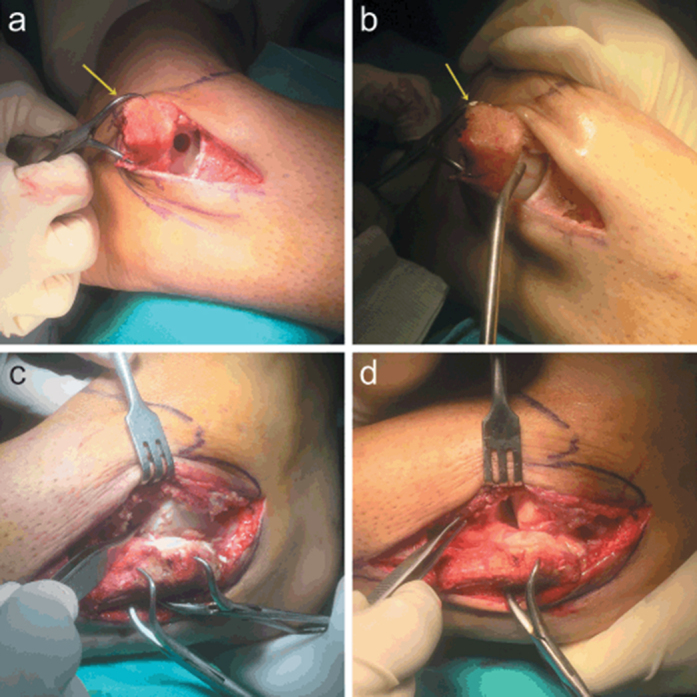
Postoperative magnetic resonance images. Twelve-month follow-up magnetic resonance images of the patient (P3) were shown as an example. Postoperative (a) sagittal T1weighted, (b) sagittal T2 weighted magnetic resonance images showing complete filling of the osteochondral defect of the medial talar dome. (c) Coronal T2 weighted and (d) sagittal T2 weighted magnetic resonance images showing complete filling of the osteochondral defect of the lateral talar dome.

All the patients were followed up at 2^nd^ week for wound check, at 6^th^ and 18^th^ weeks for radiographs and ankle functions, at 6^th^ month for clinical assessment and followed by annual follow-ups (Figure 5). Cross-bridging between the osteotomized surfaces on two orthogonal radiographs without apparent intra-articular step was considered as the radiological union in the optimal position and union time was recorded.^10^ All patients were evaluated with MRI after a year of their operation. The cartilage around the graft site was assessed with a modified Magnetic Resonance Observation of Cartilage Repair Tissue (MOCART) scoring system (Figure 6).^11^ Clinical results were graded according to the criteria of Berndt and Harty and the American Orthopedic Foot and Ankle Society (AOFAS) score.^10,12^ Tegner Activity Score and Sports Activity Level Scale (Tegner) and Lysholm Knee Scoring Scale (Lysholm) were done preoperatively, at 6 months postoperatively and the last controls.^13^ Single Assessment Numeric Evaluation (SANE) question was used to obtain patients’ subjective satisfaction with their operated ankle.^14^ Visual Analog Scale (VAS) was used to assess ankle pain at rest and during the activity.

### Statistical analysis

Statistical analysis was carried out with the paired *t*-test to compare preoperative and postoperative clinical outcomes such as AOFAS, SANE, Tegner, Lysholm and VAS scores. The statistical level of significance for all tests was considered to be 0.05. All analyses were performed using IBM SPSS Statistics Version 20.0 statistical software package (IBM SPSS Corp., Armonk, NY, USA).

## Results

Five patients with both medial and lateral OLT were treated with AOT to medial and lateral talar domes. Two of these patients (P2 and P4) had clinical complaints after previous arthroscopic BMS treatment. OLT of the other three patients (P1, P3, and P5) had a cystic lesion (stage 5) or lesion diameter ≥8 mm and lesion area ≥100 mm^2^ on MRI. Two patients (P2 and P4) had suffered a sport-related injury with inversion of the foot, and three patients (P1, P3 and P5) had experienced a similar injury while performing the daily activity. The primary complaint was pain in all patients. The average duration of symptoms from onset to osteochondral grafting was 25.2 months (range, 21-38). The average duration of symptoms from onset to first surgical intervention for two patients who had undergone unsuccessful arthroscopic treatment (P2 and P4) was 9 months and from the first operation to osteochondral grafting was 12.5 months (range, 12-13).

Preoperative patients’ data including OLT, conditions of ankle ligaments and graft sizes are shown in Table 1. There was one lesion smaller than 8 mm treated with AOT, which was a stage 5 cystic lesion. Clinical results were considered as good in all patients, according to the criteria of Berndt and Harty, and the pre and postoperative outcomes are shown in Table 2. All patients reached pre-injury levels after treatment and they did not decrease their daily living or sports activities. The mean time to return to sport was 8.2 months (range, 6-12 months). Donor site morbidity was not observed in any patients and Lysholm scores of the graft harvested knee were excellent in all patients.

The transplanted grafts were observed to incorporate fully into the recipient bed, and the radiological union was observed in all osteotomy lines on radiographs in all cases. According to MRI results a year after surgery, the articular cartilage on the tibial plafond had also healed without articular surface defects and in all cases. No decreased joint space or significant osteoarthritic changes were showed at the last follow-up radiographs. No complications were encountered.

## Discussion

Previous studies showed that OLT were more frequently observed on the medial talar dome compared to lateral talar dome.^1,3,6^ In a systemic review of 52 studies, it was shown that among 1361 patients with OLT, 62% had medial lesions, 36% had lateral lesions and only 1% had both medial and lateral lesions.^3^ Although the treatment of medial and lateral OLT separately is well established, the appropriate treatment strategy for simultaneous medial and lateral OLT is yet to be identified. We evaluated five patients with both medial and lateral OLT treated with simultaneous medial and lateral AOT in the same season. The results of our study indicate that the AOT procedure can achieve good clinical outcomes in patients with OLT in both the medial and lateral talar domes at a midterm follow-up. The AOFAS score and the SANE score significantly improved, while VAS, both at rest and during activity, significantly decreased from preoperative to postoperative. In addition, a return to pre-injury activity levels was observed, and no complications or donor site morbidity were encountered in all the patients.

In a study to evaluate the return to sports in patients with medial and lateral OLT treated with AOT separately, return to full activity was observed within an average of 6.1 months (range, 4-12 months).^15^ Similarly, Nguyen et al. stated that the average time to return to sports was 8.1 months (range, 4-18 months) after AOT in the patient with OLT.^16^ In the current study, in which medial and lateral AOT were performed in the same session, the time to return to sports was 8.2 months (range, 6-12 months), and all patients had reached their pre-injury activity levels. When return to sports after medial or/and lateral AOT are evaluated the return to sports activity were similar to those in the studies in the literature in which only medial and lateral AOT were performed.^15,16^ In addition, it can be predicted that surgery performed in a single session, as well as two separate surgical applications, reduce surgical costs and risks without creating additional complications.

The treatment of OLT is based on the patient’s previous treatment, lesion type and size of the defect and preferences of the treating clinician.^1-^^3,5^ Arthroscopic BMS is the most commonly used primary treatment of OLT.^3,4^ AOT techniques are typically reserved for large or cystic OLT, as well as failed primary repair procedures which have excellent clinical outcomes and low failure rates.^1,6,7^ Many authors utilize 8 mm as the critical-sized defect, beyond which AOT is the preferred treatment strategy.^4,5,17,18^ For large lesions (>1.5 cm^2^) and cystic lesions (stage 5), complex surgical interventions like AOT are necessary for creating hyaline or hyaline-like repair tissue.^1,2,5^ In our study, all medial talar lesions were 8 mm or larger in diameter and most of the lateral talar lesions were similar in size except one cystic lesion. Patients with cystic lesions and whose arthroscopic BMS treatment was not successful were treated with AOT. Therefore, we applied AOT to our patients with medial and lateral talar surfaces with separate medial and lateral approaches in the same session.

Simonian et al. demonstrated that the superior aspect of the lateral femoral condyle experienced less contact pressure than other articular surfaces and was the ideal donor site.^18^ In many studies, authors reported low rates of donor site morbidity of the knee after AOT.^6,7,19^ In a recent systematic review, it was reported that the total donor site morbidity rate was less than 4% and decreased over time.^7^ Hangody et al. reported that the incidence of donor site morbidity was 5%, in a multicenter case series of 354 patients who underwent AOT.^20^ In a study of 72 patients who underwent AOT, Kennedy and Murawski found a donor site morbidity only in 3 patients (4%) at a minimum 12 months follow-up.^6^

In a multicenter case series of 354 patients who underwent mosaicplasty at a mean follow-up of 9.6 years, Hangody et al. reported outcomes after mosaicplasty with multiple grafts, which demonstrated good clinical outcomes in the ankle and knee. The study also showed that the number of grafts did not affect the donor site morbidity and Paul et al. reported that the number of grafts taken does not increase donor site complications or affect functional outcomes of the knee in a single study.^17,20^ In our study, we harvested two grafts on the superior aspect of the lateral femoral condyle from the ipsilateral knees of all patients. Donor site morbidity was not observed after multiple grafts were harvested.

In a study comparing double and single plug AOT, Haleem et al. obtained similar clinic and radiological results and stated that using a double plug procedure did not show inferior clinical or radiological outcomes compared to a single plug at a minimum 5-year follow-up.^21^ Kennedy and Murawski achieved good clinical results by using the technique that they described reducing the dead space using two grafts for OLT treatment.^6^ Lee et al. concluded that the use of multiple AOT was an effective procedure for patients who present with the symptomatic stage 3 or 4 of OLT.^22^ Harvested double osteochondral plugs in these studies were transferred to the same area of the medial or lateral talar domes. However, in our study, double plugs were transferred to the medial and lateral aspects of the talar dome separately. We obtained good clinical results in all patients and excellent AOFAS scores. We believe that the placement of the double plugs separately to the medial and lateral talar domes will have similar results to placing them in the same area.

When systematic reviews of AOT procedures over the years are examined, the AOT stands out as a surgical procedure with excellent clinical outcomes as well as low complication and failure rates.^3,7,22^ An excellent clinical outcome was reported in a systematic review that showed 87.5% of the grafts presented consistency and congruity of the articular cartilage for stage 3-4 lesions.^22^ In a systematic review, nine publications described the results of 243 patients treated by AOT. Good and excellent results were obtained in 87% of patients and success rates varied from 74 to 100%.^3^ Another systematic review demonstrated that the AOT procedure resulted in good and excellent outcomes in 87.4% of patients for the treatment of OLT, with a low failure rate. The results from this study showed that patients could be reasonably counseled to expect good clinical outcomes in the mid-term. Additionally, the rate of reoperation was relatively low (6.1%), with only a small portion of these patients requiring a revision procedure or an ankle fusion (1.0%).^7^ Consistently, in our study, we showed that all patients who underwent medial and lateral AOT in the same session had excellent clinical results. There was a significant decrease in VAS score both at rest and during activity. All the patients’ AOFAS scores increased and after the treatment, the patients have reached their activity levels before the injury. Although it is very rare among ankle chondral injuries, further studies are required on larger patient groups.

In conclusion, our results showed that medial and lateral AOT can be applied simultaneously in rare cases of simultaneous medial and lateral OLT if arthroscopic BMS techniques fail or there are no suitable indications for BMS. Simultaneous AOT for medial and lateral OLT had good clinical results and provided reliable surgery. The patients successfully returned to their daily and sports activity and reported high satisfactory results in our limited case series.

**Highlights**

- Medial and lateral OLT are rarely observed concurrently and the treatment options are still being investigated.
- Simultaneous autologous osteochondral transplantation for medial and lateral OLT provides good clinical results.
- The articular cartilage of the grafted lesion appeared to be fully incorporate into the recipient bed.
- All of the patients in the study returned to their preoperative activity levels following simultaneous autologous osteochondral transplantation for concurrent medial and lateral OLT.

## Figures and Tables

**Table 1.** Patients’ Data

		Patients				
		P1	P2	P3	P4	P5
Follow-up (month)		85	77	32	78	33
	AITFL	2	1	3		3
Conditions of ankle ligaments according to MRI (grade)	ATFL	3	3	2	3	2
	DL	1	1	1	1	1
OLT distribution according to Raikin	Medial OLT	4	4	4	4	4
	Lateral OLT	6	6	6	6	6
Modified Bristol classification (stage)	Medial OLT	5	4	4	4	5
	Lateral OLT	5	4	5	4	5
Medial OLT lengths measured in MRI (mm)	Sagittal plane	15	15.9	17.1	15.1	15.9
	Coronal plane	10.3	8.7	12.2	9.4	10.7
Lateral OLT lengths measured in MRI (mm)	Sagittal plane	11.7	12.1	13.9	11.7	12.7
	Coronal plane	8.3	8.2	9.8	8.9	9.1
OLT area measured in MRI (cm^2^)	Medial OLT	1.22	1.09	1.64	1.12	1.34
	Lateral OLT	0.76	0.78	1.07	0.82	0.91
OLT area measured in the operation (cm^2^)	Medial OLT	1,1	0.72	1.2	0.64	1.1
	Lateral OLT	0.42	0.72	0.64	0.72	0.72
Graft diameter (mm)	Medial OLT	10	8	10	8	10
	Lateral OLT	6	8	8	8	8

Preoperative patient information for all the patients was summarized in the table. Conditions of ankle ligaments are classified according to MRI ligament injury grading. OLT distributions were given according to the location of the nine sites’ articular surface. Modified Bristol classification of OLT shows both medial and lateral dome OLT. Lesion lengths are measured in the sagittal and coronal plane in MRI. Lesion areas were measured both in MRI and intraoperatively. The graft diameters used in the medial and lateral talar domes are shown.

OLT, osteochondral Lesions of the Talus; AITFL, Anterior Inferior Tibiofibular Ligament; ATFL, Anterior Talofibular Ligament; DL, Deltoid Ligament; MRI, Magnetic Resonance Image.

**Table 2.** Pre-postoperative Clinical and Radiological Outcomes

		Pre-operative	Post-operative	*P*
Clinical results according to Berndt and Harty			Good	
Mean AOFAS score		35.6 (28-43)	93.4 (87-100)	< 0.001
Mean SANE score		30 (20-40)	94 (90-100)	< 0.001
Mean VAS score	At rest	7.6 (7-8)	0.8 (0-1)	< 0.001
	At during activity	9.2 (8-10)	1.2 (0-2)	< 0.001
Tegner score		5.8 (5-7)	5.8 (5-7)	
Lysholm score		100 (100)	100 (100)	
MOCART score	Medial		88 (75-95)	
	Lateral		86 (70-90)	

Preoperative and postoperative scores were given in the table for American Orthopedic Foot and Ankle Society score, Single Assessment Numeric Evaluation question, the visual analog scale at rest and during activity, Tegner activity score, and a modified Magnetic Resonance Observation of Cartilage Repair Tissue score. Paired *t*-test standard error of the difference is 0. Clinical results according to the criteria or Berndt and Harty and Lysholm knee score results were only collected postoperatively.

AOFAS, American Orthopedic Foot and Ankle Society Score; SANE, Single Assessment Numeric Evaluation Question; VAS, Visual Analog Scale; Tegner, Tegner Activity Score and Sports Activity Level Scale; Lysholm, Lysholm Knee Scoring Scale; MOCART: Modified Magnetic Resonance Observation of Cartilage Repair Tissue Score.

* Standard error of the difference is 0.
